# Decoding Sensorimotor Rhythms during Robotic-Assisted Treadmill Walking for Brain Computer Interface (BCI) Applications

**DOI:** 10.1371/journal.pone.0137910

**Published:** 2015-12-16

**Authors:** Eliana García-Cossio, Marianne Severens, Bart Nienhuis, Jacques Duysens, Peter Desain, Nöel Keijsers, Jason Farquhar

**Affiliations:** 1 Donders Institute for Brain, Cognition and Behaviour, Radboud University, Nijmegen, The Netherlands; 2 Research Development & Education Department, Sint Maartenskliniek, Nijmegen, The Netherlands; 3 Department of Kinesiology, KU Leuven, Leuven, Belgium; Scientific Institute Foundation Santa Lucia, ITALY

## Abstract

Locomotor malfunction represents a major problem in some neurological disorders like stroke and spinal cord injury. Robot-assisted walking devices have been used during rehabilitation of patients with these ailments for regaining and improving walking ability. Previous studies showed the advantage of brain-computer interface (BCI) based robot-assisted training combined with physical therapy in the rehabilitation of the upper limb after stroke. Therefore, stroke patients with walking disorders might also benefit from using BCI robot-assisted training protocols. In order to develop such BCI, it is necessary to evaluate the feasibility to decode walking intention from cortical patterns during robot-assisted gait training. Spectral patterns in the electroencephalogram (EEG) related to robot-assisted active and passive walking were investigated in 10 healthy volunteers (mean age 32.3±10.8, six female) and in three acute stroke patients (all male, mean age 46.7±16.9, Berg Balance Scale 20±12.8). A logistic regression classifier was used to distinguish walking from baseline in these spectral EEG patterns. Mean classification accuracies of 94.0±5.4% and 93.1±7.9%, respectively, were reached when active and passive walking were compared against baseline. The classification performance between passive and active walking was 83.4±7.4%. A classification accuracy of 89.9±5.7% was achieved in the stroke patients when comparing walking and baseline. Furthermore, in the healthy volunteers modulation of low gamma activity in central midline areas was found to be associated with the gait cycle phases, but not in the stroke patients. Our results demonstrate the feasibility of BCI-based robotic-assisted training devices for gait rehabilitation.

## Introduction

Stroke is the main cause of disability in adults [1]. Many patients present lower limb impairment characterized by abnormal muscle activations. Three months after the stroke, about a quarter of these patients are still bound to the wheelchair [2].

Robot-assisted training devices have been used during rehabilitation of stroke patients for regaining and improving walking ability, offering longer training duration, increasing movement repetitions and reducing the physical load imposed upon the therapist. Robotic training can provide the intensive and task-oriented type of training that has proven effective for promoting motor learning [1,3], which is thought to be useful for motor recovery after stroke [4]. Despite the lack of consensus in the literature, a recent systematic review on the topic has shown benefits of robot-assisted treadmill training. Stroke patients who received electromechanical assisted gait training in combination with physical therapy are more likely to achieve independent walking than patients receiving gait training without these devices [5].

One of the underlying mechanisms of the benefit of robot assisted-treadmill training is multisensory feedback. Multisensory feedback plays an important role in motor learning by reestablishing the sensorimotor loop that is disrupted after stroke [6,7]. Several multisensory feedback approaches have been reported for motor recovery in patients with stroke, including action-observation [8], and recently developed Brain-Computer-Interfaces (BCI) coupled to orthotic devices[9]. A BCI system can provide multisensory feedback (e.g. visual and propioceptive (robots)[10]) allowing the users to modulate their brain activity by operant conditioning [11]. BCIs can couple intention with action and enable patients with stroke to achieve intended motor actions by exploiting neural learning mechanisms [11]. Interestingly, it has been suggested that the combination of robotics and brain control of upper limb assistive technology [12–15] leads to motor learning and induces neural plasticity resulting in motor function improvement [9,16–21].

In order to develop a BCI control of the robot-assisted gait device, fundamental research aiming at detecting the precise active role of the motor cortex during the gait cycle has to be done. Furthermore, it is important to identify what can effectively and non-ambiguously be measured using non-invasive brain signals such as EEG: descending commands from the motor cortex, ascending sensorimotor information, integration of both or artifacts.

So far, only few studies have investigated the neural correlates of human walking, principally due to both the inherent experimental difficulty of measuring EEG signals in the ambulatory context and the challenging goal of balance control in walk rehabilitation tasks [22]. However, it has been recently confirmed that the motor cortex is particularly active during specific phases of the gait cycle, particularly before the foot comes in contact with the ground [23–26]. Together these studies and others [27–29] have demonstrated that supraspinal circuits, especially those of the motor cortex, have a significant role in motor control during walking. Furthermore, researchers have shown that active training can enhance motor performance and increase corticospinal excitability in comparison to passive training [30]. Therefore, for motor rehabilitation purposes it is necessary to actively involve the patients during the training.

At a fixed pattern and constant speed in robotic-training devices users often start relying on the robot to perform the movement and reduce their muscular activity [31]. An important component in the success of neural plasticity and motor learning is the supraspinal engagement during the task. Therefore, several studies have attempted at detecting active subject participation during robot training. One way to overcome this problem is by incorporating control algorithms that require the patient to actively initiate movements to perform the task. Using EEG, previous studies have investigated the difference between active and passive movement during robot-assisted gait training. A significant decrease in the mu, beta and gamma bands during active compared to passive walking was observed in the right primary motor cortex hand area, indicating increased cortical involvement during active walking [26]. However, it remains to be tested whether these cortical patterns can be classified reliably for an online detection of active cortical involvement during robot-assisted gait training.

In this study we aimed at demonstrating the feasibility of a BCI-based robotic-assisted training device for gait rehabilitation by decoding the intention of walking on the basis of EEG signals during robot-assistive gait training in ten healthy volunteers and three stroke patients with mild lower limb impairment. Moreover, we aimed at detecting the precise role of the sensorimotor cortex during active (intention to walk) and passive walking (no intention to walk) to find out to which extend the cortical involvement during gait influences the patterns of neural signals recorded by EEG.

## Materials and Methods

### Participants

10 healthy volunteers (mean age 32.3 ± 10.8, six female) without a history of neurological or psychiatric disorders and three acute ischemic stroke patients participated in the experiment (3 males, mean age 46.7±16.9, Berg Balance Scale 20±12.8). The stroke patients presented a first ischemic stroke 2, 3 and 2 months before they participated in this study, respectively. Patients presented severe left sided hemiparesis and severe difficulties to stand and walk. Detail demographic information is presented in Table 1.

**Table 1:** Demographic information stroke patients.

Patient	Age	Gender	Months after stroke	Balance Berg Score	Lesioned hemisphere	BWS	GF
P 1	51	m	2	6	right	64.20%	85%
P 2	61	m	3	31	right	35.9%	60%
P 3	28	m	2	23	right	40%	65%

m = male; BWS = Body weight support, GF = guidance force.

### Ethics Statement

The Medical Ethics Committee of the Radboud University medical center approved this study and all participants provided written informed consent before entering the study.

### Exoskeleton

**Fig 1:**
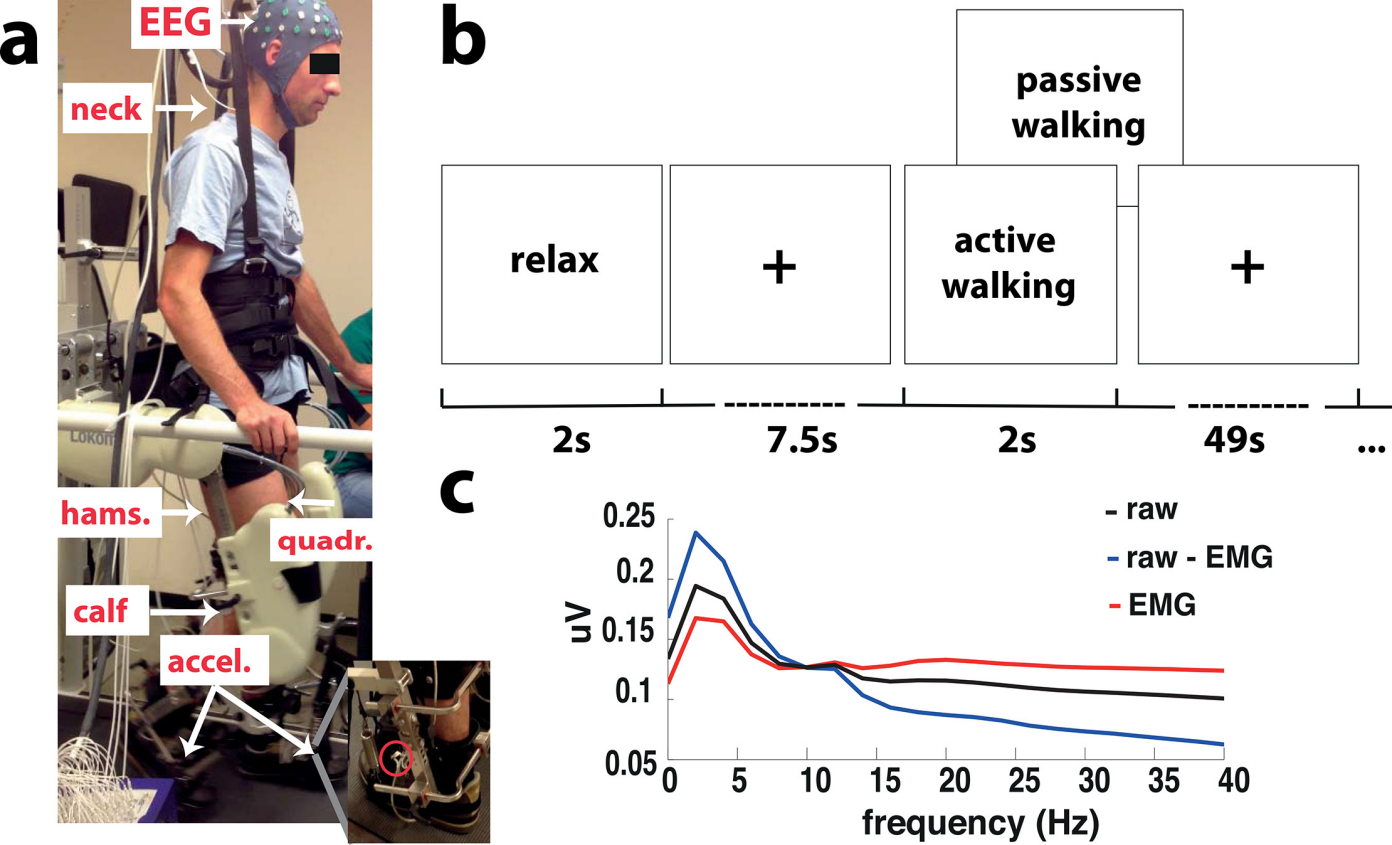
Experimental design and canonical correlation analysis example. a. Experimental setup in the lokomat with the EEG electrodes and cap, the EMG electrodes located in the right trapezius (neck), right gastroc medialis (calf), right semintendinosus (hamstr.) and right vastus lateralis (quadri.), and the corresponding accelerometers for the right and left foot (accel.). b. Schematic representation of a trial starting with a relax period hanging (baseline) and continuing with a walking task which could be either passive or active. c. Average spectrum across all blocks for all extracted components (raw signal (black)), for only the muscle related components (EMG, red) and for only unrelated muscle components (raw—EMG, blue) using canonical coherence analysis (CCA).

The Lokomat Pro (*Hocoma AG*, *Volketswil*, *Switzerland*) was used to assist walking. This exoskeleton is a bilaterally driven gait orthosis in which a body-weight support system and a treadmill are incorporated (Fig 1A). The orthosis moves the legs along a specified trajectory in the sagittal plane, with hip and knee joints of the orthosis actuated by linear drives that are integrated into an exoskeleton.

### Electroencephalographic (EEG) recordings

Electrical signals from 62 electrodes were recorded at 500Hz sampling rate using a TMSi Refa-72 amplifier (Twente Medical Systems International, The Netherlands). Impedance of the electrodes was kept below 50KΩ.

### Electromyographic (EMG) recordings

Muscle activity from the right leg (healthy volunteers) or the paretic leg (stroke patients) was recorded using three bipolar electrodes over the *gastroc medialis* (calf), *semintendinosus* (hamstring) and *vastus lateralis* (quadriceps). In addition, muscle activity from the right *trapezius* on the neck (neck) was recorded (Fig 1A).

### Accelerometers

In order to track the gait cycle during walking in the Lokomat accelerometers were positioned on each leg above the metatarsal bones (Fig 1A). The accelerometers used were type ADXL 335 (Analog Devices One Technology Way, MA, USA).

### Experimental Design

#### Healthy Volunteers

Healthy participants attended one experimental session in which they were asked to either walk in a passive or in an active mode at a speed of 1.5Km/h or to stand in the Lokomat. During the passive and active walking conditions the body weight support (BWS) and the guidance force (GF) were manipulated. For the passive walking GF of 80% and BWS of 75% were used, while for the active walking GF was set to 30% and BWS to 5% (a complete passive walking with a GF of 100% and BWS of 0% was not possible because the participants were not able to walk in the Lokomat anymore and it was not a good option either since this completely changes the stepping behavior [32]). Participants were asked to perform as little effort as possible during the passive walking condition and to allow the legs to be moved by the robot. Moreover, they were verbally informed about how passive they were walking on the basis of their EMG signals. During the task, participants hold on to the safety bars at the sides of the treadmill. Participants were instructed to follow the device as best as possible during all walking conditions (e.g. avoiding pushing against the knee and hip orthosis). A familiarization period for active and passive walking was given to the participants. After the subject felt comfortable walking in the Lokomat, the experimental session was started.

An experimental session consisted of 14 blocks, seven for passive and seven for active walking. Blocks were presented in a randomized order. Each block started with a period of quiet baseline while participants were lifted from the treadmill in the robot (100% BWS, meaning no balance needed and no contact with the floor) and looking at a fixation cross on a computer screen for 7.5s (Fig 1B). Afterwards an instruction was displayed on the screen advising the participant about the task to be executed; active or passive walking. In both walking tasks, the treadmill started, participants walked for 49s, after which the treadmill was stopped. The treadmill had a delay of at least 7s to come up to a stable speed and 7s to slow down and stop completely. The recordings only took place during constant and stable speed, which was indicated by the physiotherapist controlling the robotic device and a trigger marker in the EEG recordings. During the baseline and walking periods a fixation cross was displayed on the screen. Resting periods between blocks were made depending on the participant’s fatigue.

#### Stroke patients

Stroke patients also attended one experimental session in which they were asked to walk at a comfortable speed of maximum 1.5Km/h or to rest in the Lokomat with 100% of BWS and no contact with the floor (baseline). A physiotherapist controlled the speed of the orthosis according to the patient’s capabilities. The BWS and the GF was adjusted for each patient’s limitations (see Table 1).

For patients, an experimental session consisted of 10 blocks. Each block started with a period of lifted from the treadmill (100% BWS, meaning no balance needed and no contact with the ground) while participants looked at a fixation cross on a computer screen for 7.5s, which was used as a baseline condition (Fig 1A). Subsequently, an instruction was displayed on the screen advising the participant about the initiation of the task. During walking, the treadmill started and patients walked for 49s, after which the treadmill was stopped. The physiotherapist indicated when the patient walked properly. During the baseline and walking periods a fixation cross was displayed on the screen. Resting periods between blocks were made to avoid fatigue.

### EEG analysis

EEG data were downsampled to 250Hz, linearly detrended and epoched according to gait cycle information recorded from the accelerometers. The gait cycle phases were defined relative to the right heel strike (measured by the accelerometers) for the healthy volunteers and for the patients relative to the paretic leg heel strike (see S1 Fig). The other gait cycle phases, apart from the right heel strike, were defined according to the literature [33]. In total, around 17 to 21 gait cycles were detected in each block, depending on the participant’s leg length. EEG signals were re-referenced to a common average across all channels. A Canonical correlation analysis (CCA) method [34] was used to remove the EMG artifacts on the EEG signals. This worked by identifying and removing sources (components), such as muscle activity, which have low temporal auto-correlation as assessed by having power in the EMG frequency band (15-30Hz) more than 1.3 times stronger than in the EEG frequency band (1-30Hz). The components identified as muscle activity are marked as EMG and removed from the raw signals. The remaining components are kept and used to reconstruct the EEG activity (Fig 1C). The mastoid electrodes located on TP8 and TP7 were removed and on the remaining EEG electrodes a surface Laplacian based on spherical spline interpolation [35] was performed to improve spatial selectivity.

Power spectral analysis was performed using Welch’s method with a Hanning window of 250ms. For classification of the EEG signals into different conditions, the frequency bins from 8 to 30Hz were used. Frequencies below 8 Hz were not considered in the analysis in order to avoid any influence from movement artifacts (<4Hz).

Event-related desynchronization (ERD) and event-related synchronization (ERS) were calculated by normalizing the power in the frequency of interest from the active and passive walking by the corresponding baseline condition. The following equation (Eq 1) illustrates this procedure:

$$\text{ERD or ERS}=\left(\frac{Walking-Baseline}{Baseline}\right)\times 100$$ (1)

Walking corresponds either to passive or active walking and the Baseline to the resting period before passive and active walking, respectively.

Spectrograms for the active and passive walking and baseline were calculated for each trial using a hanning window of 250ms and a fractional overlap of 0.5 and afterwards time-warped such that all gait cycles had the same effective duration. Event-related spectral perturbations (ERSP) were calculated by first subtracting the average power over the gait cycle from the power at any time-point and then dividing by the average power over the whole experiment for each frequency bin.

### Classification of EEG signals

EEG classification was performed using a L_2_-regularized logistic regression classifier [36,37]. The regularization parameter was selected as the one, which maximised the estimated classification accuracy. The EEG epochs were classified between passive walking and baseline, active walking and baseline and passive and active walking (binary classification). Classification accuracy was estimated using 10 fold cross validation on the testing data, where for each fold 90% of the trials were used for training the classifier and 10% for testing it. The output of the classifier for each fold was the prediction decision values for each instance. Classification accuracy was measured by the balanced loss [38], which punishes stronger a wrong classification of an instance from the minority class than a wrong classification of an instance from the majority class (this to prevent for unbalanced number of trials for each class). This value falls in the range of 0 to 1 and after multiplying it by 100(%), an accuracy of 50% represented the chance level or no discrimination.

The classifier weight vectors for the best regularization parameter were calculated for each participant in order to identify which frequency and spatial characteristics were most useful for the classifier. A grand-average classifier weight vector was obtained across participants by normalizing each participant’s classifier weight vector. Values between 1 and -1 were obtained, where values close to 1 and -1 indicated a strong influence on the classification and values close to zero indicated no influence on the classification.

### EMG analysis

EMG signals were filtered with a high-pass filter in 10Hz and a notch filter was applied to remove the power line noise in 50Hz. Subsequently, signals were normalized to the maximum value during baseline, then rectified, downsampled to 250Hz and epoched according to gait cycle information recorded from the accelerometers (as it was done with the EEG data). Grand average muscle activity was calculated first by normalizing the EMG signals across muscles and walking conditions for each participant and then averaging. Normalized EMG signals varied from 0 to 1, where 1 indicated the maximum activity across muscles and across walking conditions for each participant.

### Statistical analysis

All data were reported as mean values ± standard deviation (SD) when indicated. Statistical evaluations on the power density analysis were performed using a paired sample t-tests.

A cluster-based permutation test [39] was used to assess differences on the ERSP across moving conditions (active, passive walking and baseline). This non-parametric test finds clusters of frequencies and time points where the spectrogram differs between conditions while controlling for the false alarm rate. Cluster-based permutation tests were performed using Fieldtrip [40]. The significant level (α) was 0.05.

Significant differences in muscle activation patterns were calculated between active and passive walking. For this purpose the EMG signals for each muscle were normalized to the maximum value during baseline and downsampled to 250Hz. Then, a power spectral analysis was performed using Welch’s method with a Hanning window of 250ms on the signals from each muscle. For group analysis the power for each muscle was normalized to the maximum for each subject. EMG grand average was calculated independently for each muscle. A pair sample t-test was used to compare each frequency bin (from 0 to 40 Hz) from the EMG grand average during active walking against passive walking. The significant level (α) was 0.05 for all tests. Bonferoni correction was applied to correct for multiple comparisons among frequencies (p = 0.0013, corrected).

As classification accuracies are not normally distributed [41] a non-parametric test, Wilcoxon signed rank, was used to assess significant classification accuracies above chance level. To compare the classification performance across decoding conditions a non-parametric one-way ANOVA (Kruskal-Wallis) was performed. Post-hoc Wilcoxon signed rank tests were performed for subsequent comparisons. The significant level (α) was 0.05 for all tests. Bonferoni corrections for multiple comparisons were applied when required.

## Results

### Healthy volunteers task

#### Canonical correlation analysis (CCA)

Since physiological signals like muscle artifacts can contaminate strongly the electroencephalographic (EEG) signals during walking, a canonical correlation analysis (CCA) was performed. CCA component analysis rejection method has shown effectiveness in removing the EMG components from the EEG signals [42] (for an example see 1c). This analysis was fed time-delayed versions of the signals such that it could derive spectral as well as spatial filtering. Before rejection of the EMG components the raw EEG data contains strong power in frequencies in the EMG range (above 15 Hz), which is spatially clustered around the known muscle sources, i.e. neck, eyes and scalp muscles. After rejecting the EMG components and selecting correctly the EEG components the power on these frequencies and locations is reduced. Therefore, after CCA analysis the EEG signals are cleaned from EMG components that might bias the classification results (based on non-cortical signals).

#### Power density analysis and topographical distribution

**Fig 2:**
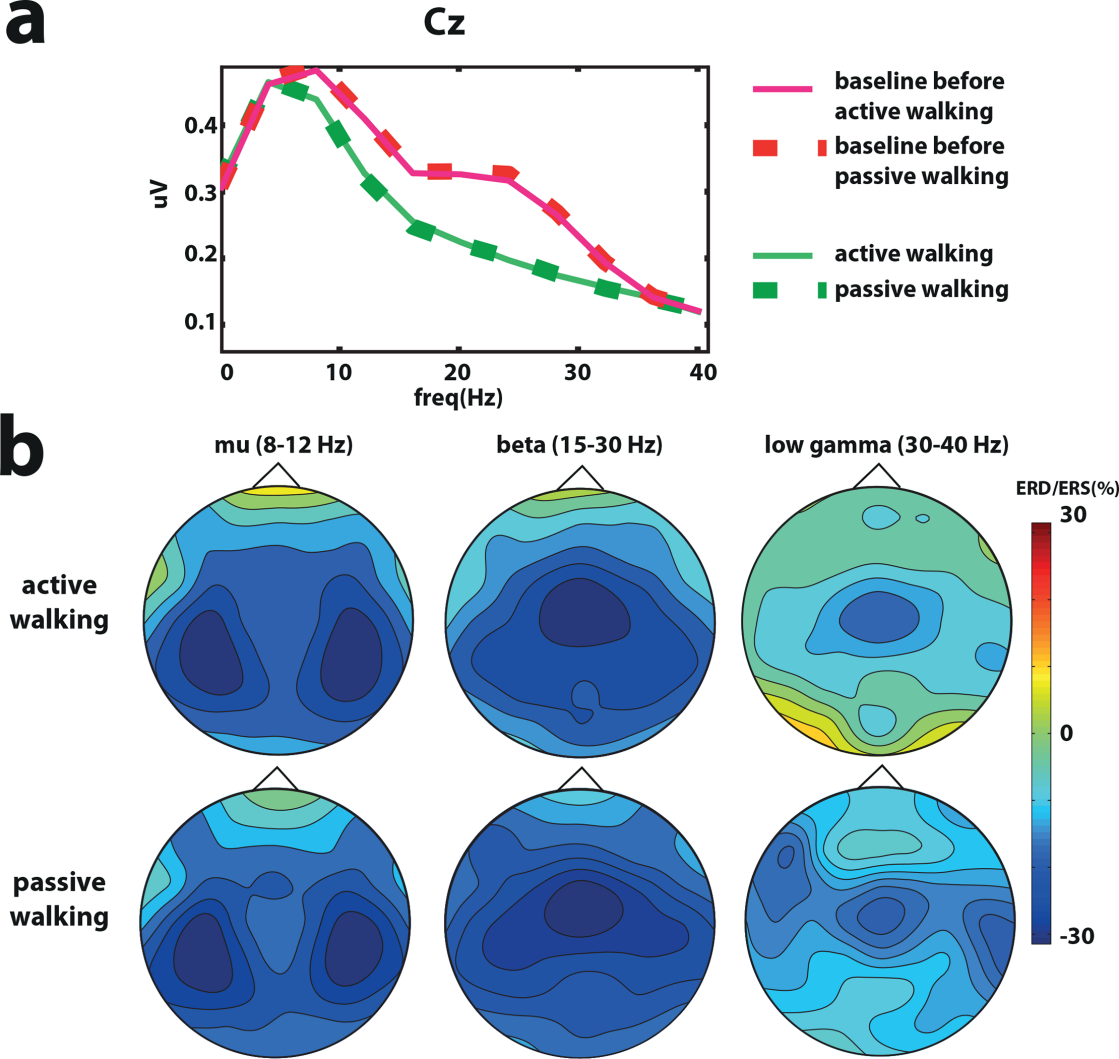
Power density analysis in healthy volunteers. a. Grand average power density analysis over Cz for active and passive walking and the baseline before passive and active walking conditions. b. Topographic distribution of event related desynchronization (ERD) and synchronization (ERS) in the mu (8–12 Hz), beta (15-30Hz) and low gamma (30-40Hz) bands.

A significant difference between passive walking and active walking and its corresponding baseline condition were found in the beta band in the Cz electrode (Fig 2A) (active walking vs baseline before active walking t = 2.15 p = 0.04, passive walking vs baseline before passive walking t = 2.21 p = 0.04). Event related desynchronization (ERD) was stronger bilaterally above the right and left primary motor areas in the mu band (8-12Hz), while in the beta (15-25Hz) and low gamma (30-40Hz) frequencies the ERD was stronger above the Cz electrode in the fronto-central leg motor area (Fig 2B).

#### Spectrogram

**Fig 3:**
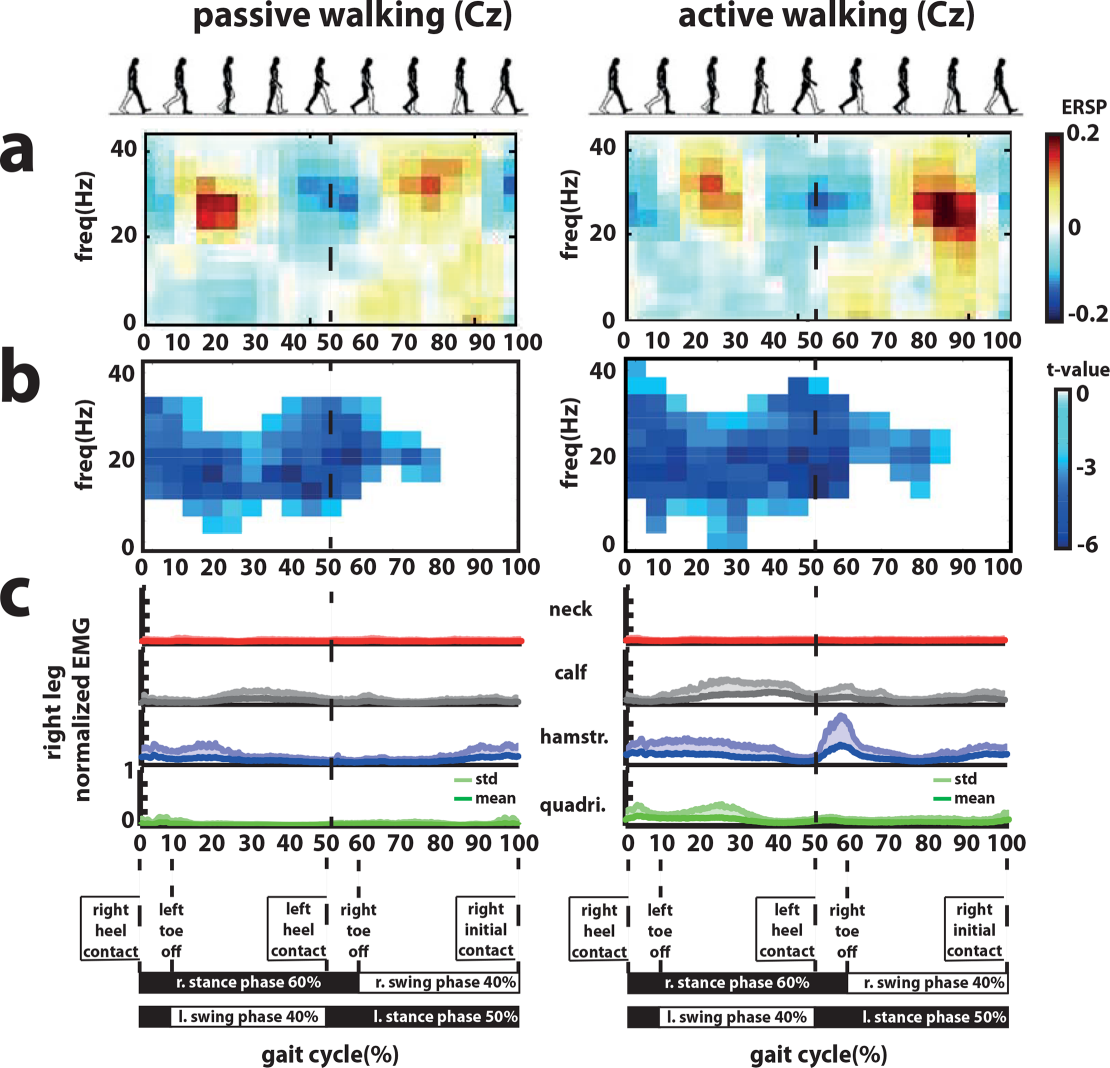
Event-related spectral perturbations (ERSP) and muscle activity during the gait cycle in healthy volunteers. a. Grand average event-related spectral perturbations (ERSP) over Cz during active and passive walking. b. Statistical differences between passive walking and baseline (left) and active walking and baseline (right). c. Grand average muscle activity (normalized across muscles and walking conditions) from the neck (right *trapezius)*, right calf (*gastroc medialis*), right hamstring (*semintendinosus*, hamstr.) and right quadriceps (*vastus lateralis*, quadri.).

Grand average ERSPs for the channel Cz showed clear changes in the spectral power during the gait cycle in the high beta and low gamma band (20-40Hz) (Fig 3A). Between the right initial contact and left toe off phase a desynchronization was found in high beta and low gamma band. The same pattern was observed during left initial contact and right toe off phase. On the contrary, during left and right swing phases (after left and right toe offs until right and left initial contact) a synchronization in the high beta and low gamma band was found (Fig 3A).

The cluster-based permutation t-tests of the EEG signals from Cz revealed significant differences in the ERSPs between (active and passive) walking and baseline conditions (Fig 3B). Passive and active walking had mainly a significant decrease in power in the frequency range of beta and low gamma frequencies. No significant differences were found when the cluster-based permutation t-test was performed between active and passive walking conditions.

#### Muscle activity

Grand average EMGs are shown in Fig 3C for passive and active walking. The *calf* was active during the right stance phase after the left toe off. The *hamstring* was activated directly after the right heel contact during the right stance phase and also after the left heel contact and right toe off phase. The *quadriceps* was active between the 20% and 30% of the gait cycle during the right stance phase. No systematic changes in muscle activity during the gait cycle were observed in the neck muscle.

**Fig 4:**
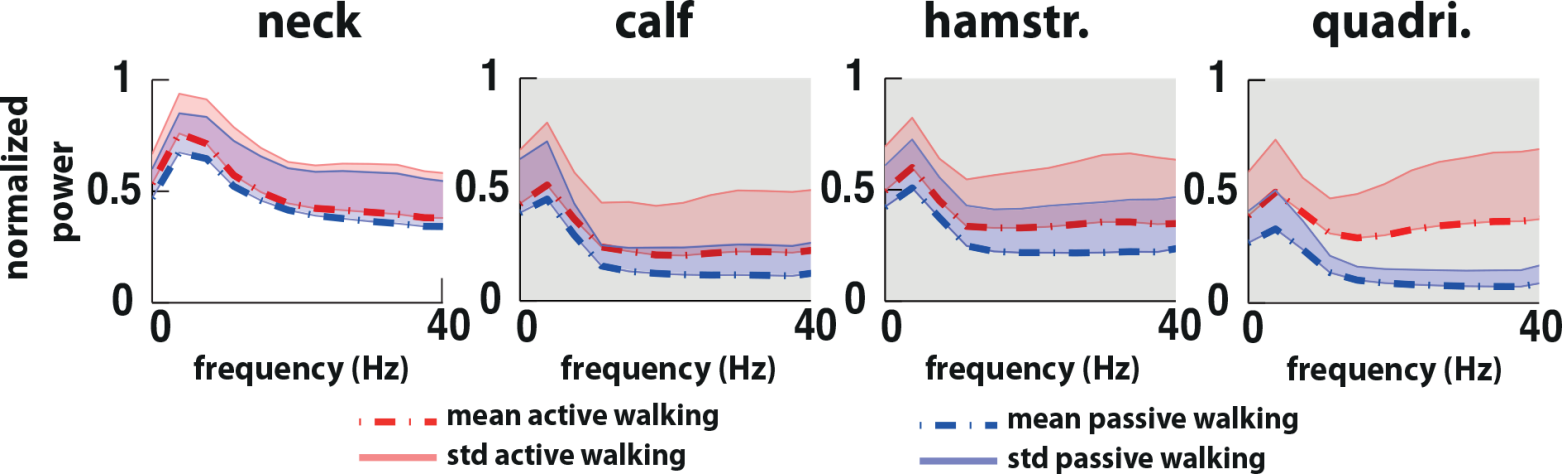
Power spectrum EMG grand average. Power spectrum grand average for the neck (right *trapezius)*, right calf (*gastroc medialis*), right hamstring (*semintendinosus*, hamstr.) and right quadriceps (*vastus lateralis*, quadri.) during active (red) and passive (blue) walking. Statistical differences between passive walking and active walking are highlighted in the gray shadow (p<0.0013, corrected).

Significant differences (p<0.0013 corrected) in muscle activation patterns indicated that during active walking muscle activity in the right lower limb was increased in comparison to passive walking. The later occurred in all the frequencies between 0 to 40Hz (Fig 4). The neck muscle did not show any significant difference between passive and active walking.

#### Classification accuracies

The average classification accuracy was 94.0±5.4% (mean±std) when active walking was compared against baseline and 93.1±7.9% when passive walking was compared against baseline (Table 2). A classification performance of 83.4±7.4% was achieved when active walking was compared against passive walking. Furthermore, when the baseline before active walking and the baseline before passive walking were compared the classification performance was 54.7±8.3% (not significantly different from chance level). All comparisons showed classification performances above chance level (50%) (active walking vs baseline p<0.01 corrected (p = 0.002); passive walking vs baseline, p<0.01 corrected (p = 0.002); and active walking vs passive walking p<0.01 corrected (p = 0.002)) except when the baseline before active walking and the baseline before passive walking conditions were compared (p>0.05 corrected (p = 0.13)), as expected.

**Table 2:** Classification performance for different walking conditions (active and passive) and baseline.

participants	active walking vs baseline	passive walking vs baseline	active walking vs passive walking	baseline before active walking vs baseline before passive walking
**1**	94.9	95.5	89.9	42.6
**2**	97.4	98.8	75.3	43
**3**	100	100	83.3	63.7
**4**	85.3	85.3	83.1	53.6
**5**	98.6	100	77.5	60.5
**6**	96.6	92.6	79	46
**7**	95.7	94.5	91.7	66
**8**	93.3	93.3	93.7	58
**9**	94.3	96.6	88.3	56.8
**10**	83.5	74.5	71.9	57.3
**mean**	94	93.1	83.4	54.7
**std**	5.4	7.9	7.4	8.3

The non-parametric one-way ANOVA showed a main effect between classification accuracies (χ ^2^_3,39_ = 28.33, p = 3.1x10^-6^). Post-hoc Wilcoxon signed rank tests indicated that the classification performance of active and passive walking against baseline had a higher classification performance than the classification performance when active walking was compared against passive walking (p<0.01 corrected (p = 0.002) for active walking; p<0.01 corrected (p = 0.002) for passive walking) and the baseline’s classification task (p<0.01 corrected (p = 0.0020) for active walking; p<0.01 corrected (p = 0.0020) for passive walking). No differences were found when the classification performance of active walking vs baseline and passive walking vs baseline were compared (p>0.05 corrected (p = 0.92)).

**Fig 5:**
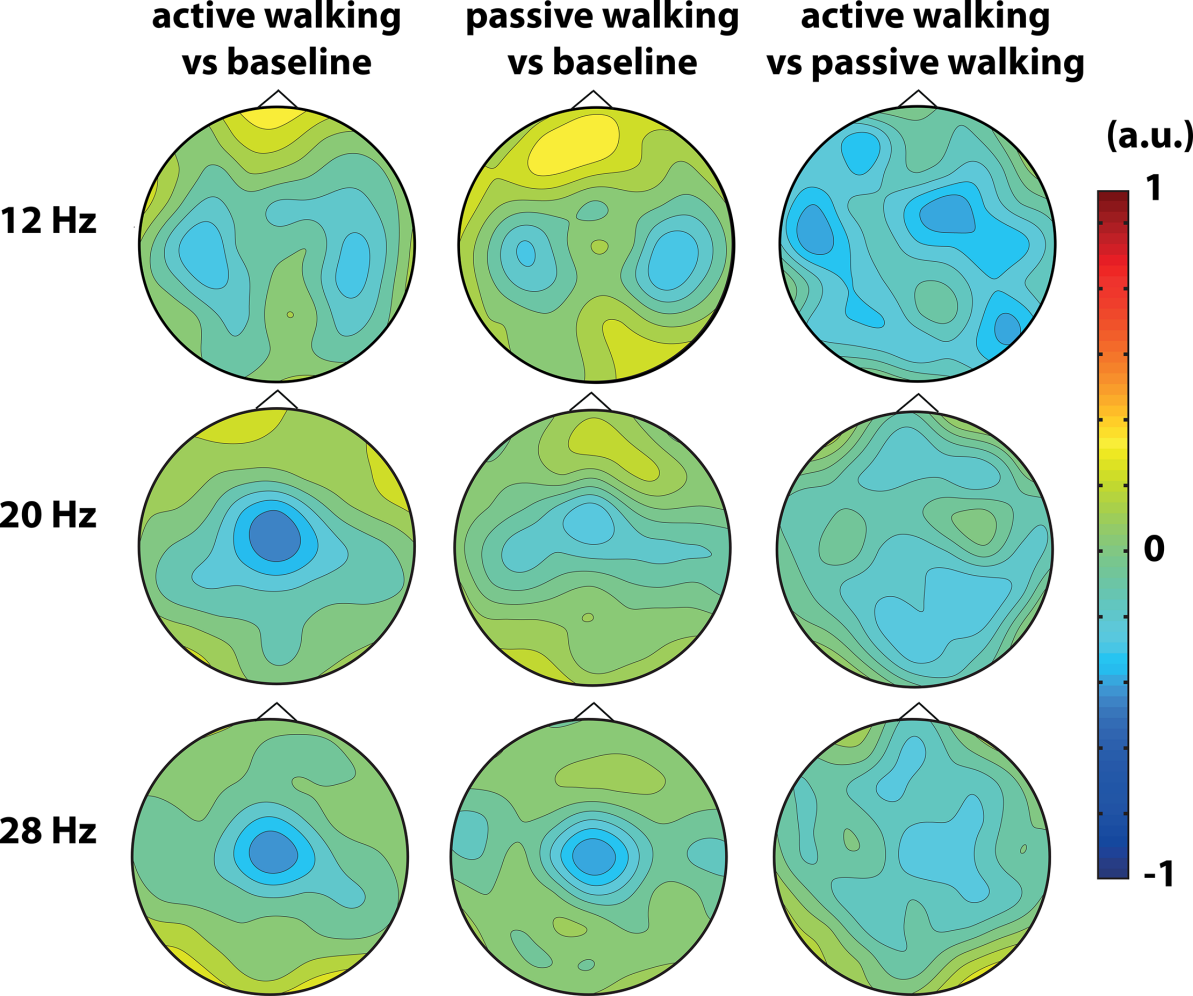
Classifier weight vector in healthy volunteers. Grand average classifier weight vector for the best regularization parameter representing frequency (12Hz, 20Hz and 28Hz) and spatial characteristics used by the classifier.

The grand average classifier weight vector (Fig 5) for active walking against baseline and passive walking against baseline showed features corresponding to the ERD which could be seen at the expected channels around bilateral motor cortices for the mu frequency (12Hz) and more medial around Cz for the beta (20Hz) and low gamma frequencies (28Hz). For active walking versus passive walking, a weaker brain activity was found around bilateral motor cortices in the mu frequency (12 Hz) while in the beta band this activity was localized more in parieto-occipital areas and in the low gamma band in centro-medial areas.

The grand average classifier weight vector (Fig 4) for active against passive walking showed features corresponding with weaker mu strength in pre-motor regions, weaker beta strength in the pre-frontal and posterior parietal regions and weaker gamma strength in the leg motor cortex area during active robot-assisted walking. However, pair sample t-tests indicated no significant difference between passive and active walking in the electrodes and frequencies where the classifier weights were stronger (12 Hz channel P04 t = -1.41 p = 0.19 and channel FC2 t = -0.33 p = 0.75; 20Hz channel POz t = -1.13, p = 0.28 and channel Fz t = 1.51 p = 0.17; 28 Hz channel C2 t = 1.59 p = 0.15).

### Stroke patients

Here the results obtained for the stroke patients are described.

#### Power density analysis and topographical distribution

**Fig 6:**
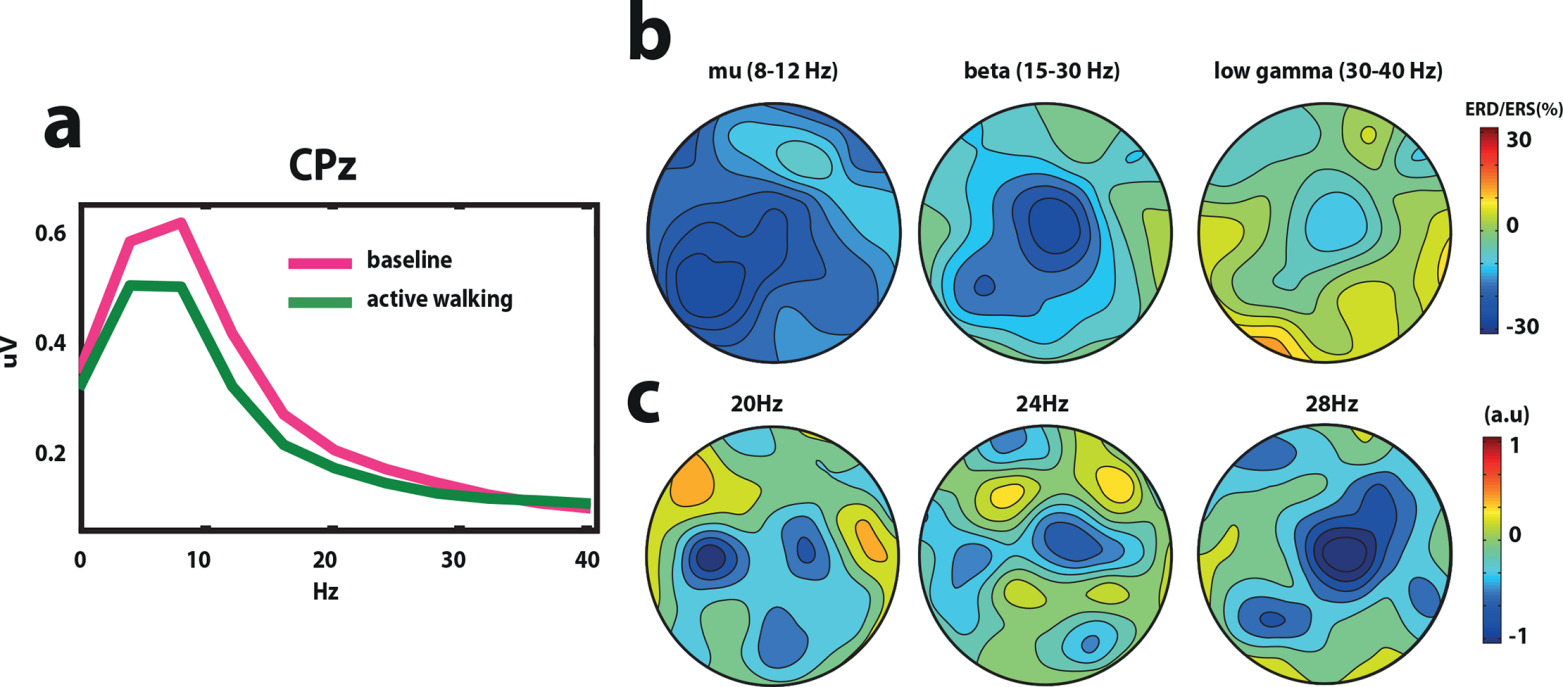
Power density analysis and classifier weight vector in stroke patients. a. Grand average power density analysis over Cz for baseline and walking conditions. b. Grand average topographic distribution of event related desynchronization (ERD) and synchronization (ERS) in the mu (8–12 Hz), beta (15-30Hz) and low gamma (30-40Hz) bands. c. Grand average classifier weight vector for the best regularization parameter representing frequency (20Hz, 24Hz and 28Hz) and spatial characteristics used by the classifier.

A significant difference between active walking and the baseline condition was found in the beta band (25–30 Hz) in the CPz electrode (t = -5.87 p = 0.03) (Fig 6A) in the stroke patients. Event related desynchronization (ERD) was found stronger distributed over the left centro-parietal areas in the mu band (8-12Hz) (towards the healthy hemisphere). In contrast, in the beta (15-25Hz) and low gamma (30-40Hz) frequencies the ERD was stronger above the Cz electrode in the fronto-central leg motor area and moderate stronger above the left centro-parietal area (Fig 6B).

#### Spectrogram

**Fig 7:**
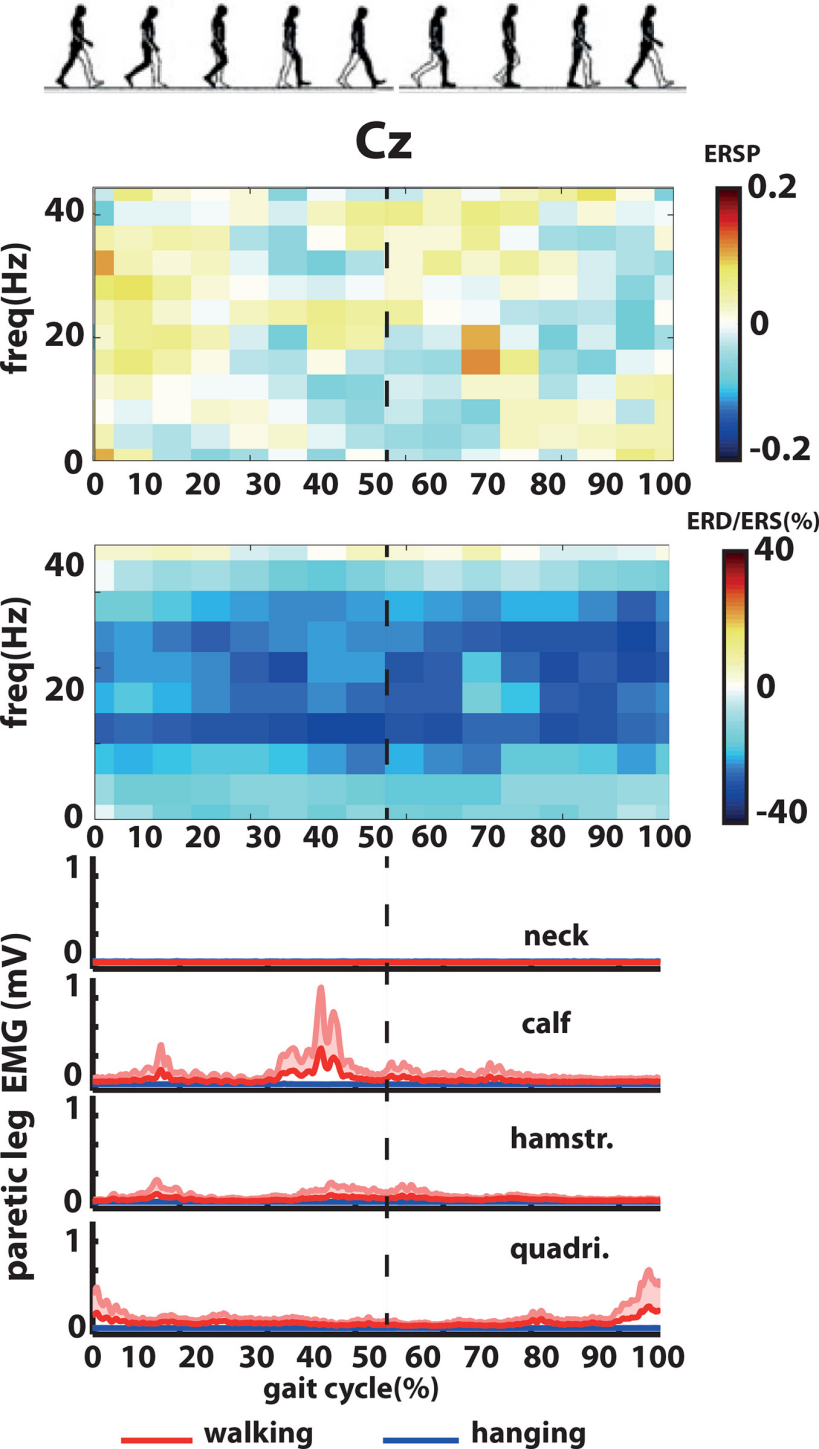
Event-related spectral perturbations (ERSP) and muscle activity during the gait cycle in stroke patients. a. Event related spectral perturbations (ERSP) over Cz during walking. b. Event-related desynchronization and synchronization (ERD/ERS) during walking (no threshold for significant changes was applied). c. Muscle activity from the neck (right *trapezius)*, right calf (*gastroc medialis*), right hamstring (*semintendinosus*, hamstr.) and right quadriceps (*vastus lateralis*, quadri.) during walking (red) and baseline (blue).

ERSPs for the channel Cz (Fig 7A) did not show systematic changes during the gait cycle in the spectral power as found in healthy volunteers in the low gamma band (Fig 3A). The cluster-based permutation t-tests of the EEG signals from Cz did not revealed significant differences between the ERSPs during active walking and baseline. However, ERD/ERS from Cz revealed a strong desynchronization during walking in the beta band (15-30Hz) (Fig 7B).

#### Muscle activity

Grand average EMGs are shown in Fig 7C for baseline and active walking. EMG activity of the stroke patients showed a different muscle activation pattern during the gait cycle when compared with the healthy volunteers’ pattern (Fig 3C). The activity in the *calf* was more pronounced during the right heel contact and before left heel contact during the right stance phase. In the *hamstring* the activity was increased directly after the left toe off phase until the left heel contact during the right stance phase. The *quadriceps* activity was increase before the left heel contact (between the 40% and 60% of the gait cycle during the right stance phase). No systematic changes in muscle activity during the gait cycle were observed in the neck muscle.

#### Classification accuracies

The classification accuracy was 96.4%, 85.7% 87.5% for patient 1, 2 and 3, respectively when walking was compared against baseline using the information from the beta band (20–30 Hz). The grand average classifier weight vector (Fig 6C) shows features corresponding to the ERD (Fig 6B), which can be seen at the expected channels around Cz for 20, 24 and 28Hz.

## Discussion

The main aim of this study was to demonstrate that EEG based BCI could be used for the control of a robot-assistive gait device, despite the presence of prominent muscle and movement artifacts during walking. In order to demonstrate this we evaluated: the performance of a linear classifier to differentiate between walking intention and no walking intention in healthy volunteers, the cortical involvement during walking intention and its topographical distribution in the sensorimotor stream, and the performance of this classifier in stroke patients with locomotor deficits.

Classification performances differentiating walking from baseline for both healthy participants and stroke patients were above 93% and 89%, respectively, supporting previous results during treadmill walking [25]. This underlines the feasibility of BCI-based robotic-assisted training devices. The weights of the classifier indicated that the main brain signals contributing to this performance were the event-related desynchronization (ERD) in the mu rhythm more bilaterally distributed and ERD in the beta and low gamma bands located more centro-medially as has been reported before [25,42]. Brain signals in the beta band centro-medially located (Cz electrode) were significantly different during passive walking and active walking in comparison to baseline. We observed that mu-band effect seemed more lateralized over the hand areas whereas beta/gamma was more medially focused over the foot regions. This could be due to implicit hand activity or volume conduction effects causing central mu-cancellation. Previous NIRS studies have measured cerebral activity during actual gait [27,43], showing that walking increases cerebral activity bilaterally in the medial primary sensorimotor cortices, the supplementary motor area and the prefrontal cortex. Furthermore, successful conversion of brain signals during walking from the motor cortex into limb kinematics has been achieved with monkeys, on the basis of invasive measurements [29]. Our evidence is in accordance with these studies indicating that supraspinal circuits have a significant role in motor control during walking.

Furthermore, we aim at detecting the precise control role of the sensorimotor cortex during active (intention to walk) and passive walking (no intention to walk) in order to find out to what extend the cortical involvement during gait influences the patterns of neural signals recorded by EEG. In the first place, we found significant stronger muscle activations during active walking in comparison to passive walking indicating that the healthy controls were able to relax and not contract voluntarily their muscles during passive walking as during active walking. However, it is difficult to avoid active muscle contraction in the legs during “complete” passive walking because it is hard not to resist the imposed movements at times and as soon as some load bearing is allowed some muscle activations are present (e.g. in the soleus) [44]. This explains why during passive walking we still found some voluntary muscle contraction. In the second place, a good classification performance was found when distinguishing passive and active walking (above 83%) in the healthy volunteers. The weights of the classifier indicated differences in brain activations in pre-motor, posterior parietal and in the leg motor areas (in the mu, beta and low gamma rhythms, respectively) between active and passive robot-assisted walking. However, these differences were not statistically significant. We conclude that the classifier is picking up information that is not localized to a specific frequency or region in the brain, but it is driven by broadly distributed signals. Even tough, we found that the ERD in central-middle areas (i.e. efferent signal) were driving the classification between active and passive walking against rest, one cannot completely reject that afferent sensory information was also identified by the classifier. This might be one of the reasons why the classifier reduced in performance when active walking was classified against passive walking and no significant differences were found between these two walking conditions. Therefore, for future implementations into BCI control one has to consider that this approach cannot differentiate between walking intent, simply sensory information emerging from locomotor activity or a mix of these two signals. Furthermore, despite applying CCA, remaining sources of unwanted artifacts cannot be entirely excluded and therefore they could have potentially contributed to the classification between passive and active walking

In correspondence with previous studies [26] a modulation of the high beta and low gamma activity during the gait cycle was observed in the event-related spectral perturbation (ERSP) analysis during active and passive walking. We were able to detect the difference between left and right swing and double support phases without using ICA and source analysis techniques. This modulation showed an ERD during the double support phase (when muscles are less active) while during the swing phase (when muscle are more active) an ERS, which might imply more cortical involvement during the double support phase. Moreover, compared with Wagner’s et al. [26] data, during passive walking the present data shows similarities for the swing phase but differs for the stance period in that more activity is seen in the present data. One likely explanation is the difference in BWS between both studies during passive walking (in Wagner et al. [26] BWS was always below 30% while in the present study it was 75%).

Other studies have found ERSPs in a broader band including not only low gamma and high beta activity but also mu and low beta modulation coupled to the gait cycle during treadmill walking [23,25,42]. One possible explanation because this modulation was so focal in the high beta and low gamma band in our experiment could be because of the fixed walking pattern imposed by the robot (less variation), which is different than when people walk more naturally in a treadmill. Previous work has suggested that the sensorimotor system may shift towards operating at higher frequencies (gamma) in situations requiring dynamic force output [45]. Another possible explanation is based on previous work [46] showing that movement artifacts were associated with broader low gamma modulation of the ERSP. Since our results showed ERSPs that on the contrary were frequency specific to high beta and low gamma activity (between 20 to 40Hz), this could actually suggest that our data were less prone to movement artifacts (which were found mainly in the low frequency bands below 4 Hz and not in the beta and low gamma frequency) (See S2 and S3Figs).

Importantly, we did not find the modulation of the high beta and low gamma activity during the gait cycle in the small population of stroke patients that we assessed. Gamma synchronization facilitates the coordination of distributed functional cell assemblies [47] and is a fundamental process in cortical computation [48]. Seeber et al. [24] suggested that low gamma oscillations of neuronal populations might be linked to sensorimotor processing or integration. Even though it is difficult to conclude anything regarding only three patients, we hypothesize that the lack of low gamma modulation during the gait cycle might be related to the sensorimotor integration deficits presented in these stroke patients. Future studies should investigate whether modulation of low gamma frequencies before and after robot-assisted gait training could be used as a potential biomarker of motor recovery.

Even though in this study we used participants trying actively to match the movement of the Lokomat as a model of walking, it is important to underline that during Lokomat walking the kinematic patterns are slightly different than during treadmill walking (e.g. more hip and ankle extension, greater hip and ankle range of motion and less linear movement of joints)[49]. These differences might modify the type of motor commands send by the cortex. However, for the purposes of detecting walking intention this model is sufficient.

Our current classification results (above 89%) are relevant for developing a BCI-based robot-assisted gait training device controlled by EEG signals. Although previous studies have shown that it is feasible to remove the EMG artifacts from EEG signals recorded during walking using ICA and dipole fitting [26], these methods are not so straightforward to be implemented in an online BCI scenario due to their computational load. Instead, canonical correlation analysis (CCA) has shorter computational time and can be used on a trial basis as others have shown [50]. Therefore, CCA can be easily implemented during online BCI.

Previous studies have shown already the feasibility of an online control of gait rehabilitation devices driven by EEG signals [22]. For example in [51], a non-invasive EEG-based BCI governing a functional electrical stimulation (FES) system for ankle movement was presented. In this application, EEG patterns underlying foot dorsiflexions were detected in real time, and this information was subsequently used to trigger the FES. A linear Bayesian classifier trained using a vector of spatio-spectral features optimally discriminated the idling and dorsiflexion states. In relation to our results, EEG power changes in the μ, β and low γ bands observed over mid-central areas (i.e., electrode Cz) were the most informative features for classification. In addition, all five able-bodied subjects achieved a 100% BCI-FES response (no omissions), and one subject had a single false alarm. In another study [52], paraplegic and tetraplegic patients could trigger a walking simulator (virtual reality) by imagining themselves walking or idling. In a follow up study [53], one able-bodied subject and one subject with paraplegia due to spinal cord injury (SCI), used an EEG prediction model for online BCI operation of the Lokomat. The EEG in the pre-frontal cortex, supplementary motor, and the leg and arm sensorimotor representation areas contained the best discriminant information, which is in line with our results regarding the classifier’s topographic weight information. The cross-correlation between instructional cues and the BCI-Lokomat walking epochs averaged was 0.81±0.05 (0.8 false alarms per session and no omissions). This proved that SCI patients have the possibility to operate a robust BCI walking simulator with a short training period and satisfying accuracy. Our results are in line with these studies performed in SCI, showing that EEG detected movement intention can be effectively used for a BCI-gait-rehabilitation system also in patients with cortical stroke.

Previous studies have shown that a binary control (on/off) of a BCI-based robotic device in combination with physiotherapy has a beneficial effect in the motor rehabilitation of the upper limb of stroke patients with severe paralysis [9]. However, it remains to be tested if such BCI might benefit the motor rehabilitation of the lower limb. In order to develop a BCI for the rehabilitation of gait, it is necessary to evaluate the feasibility to decode walking intention from cortical patterns during robotic lower limb rehabilitation. Our offline classification results showed that it is feasible to distinguish with high accuracy between resting and walking when stroke patients are immersed in a robot-gait training system. In previous work [54], we have demonstrated that the online implementation of this approach (i.e. using a logistic regression classifier to distinguish walking intention against resting) can be used to control in a binary mode (on/off) a treadmill by using EEG signals from healthy volunteers, achieving high accuracies rates [37]. All together, these results showed the feasibility of developing a BCI for the rehabilitation of gait.

Lastly, one problem of brain control approaches is that the accuracy to detect intention or movement by noninvasive brain signals can be limited [55–58]. On the other hand, surface electromyography (sEMG) activity has been successfully used for the accurate decoding of many movements for prosthesis' and orthosis’ control of the upper [59–62] and lower limb [63], making it an attractive tool as a source of control for motor restoration robotics or orthotics.

## Conclusions

Here, we demonstrated that it is possible to decode walking intention from cortical patterns generated in the sensorimotor strip during robot-assisted gait training in healthy volunteers but also in stroke patients with mild lower limb impairment. The modulation of low gamma activity in central midline areas was found to be associated with the gait cycle phases in healthy volunteers but not in the stroke patients.

## Supporting Information

S1 File Supporting Information. (PDF)

S1 Fig Gait cycles were determined according to the right heel strike (red dots) using the accelerometer’s data from the right (blue continue line) leg. The left leg’s accelerometer data is as well illustrated (black dotted line) for comparisons.
(TIF)

S2 Fig Power density analysis of EMG artifacts in healthy volunteers. a. Spectral density analysis over all electrodes for active and passive walking and the baseline before passive and active walking conditions. b. Topographic distribution of event related desynchronization (ERD) and synchronization (ERS) in the mu (8–12 Hz), beta (15-30Hz) and low gamma (30-40Hz) bands.
(TIF)

S3 Fig EEG temporal structure (in Cz) during the gait cycle for one of the participants. From top to bottom: average across trials of raw data, EEG data filtered in the beta band (15–30 Hz), EEG data filter in the low gamma band (30–40 Hz) and event-related perturbations (ERSP) from 0 to 40Hz for active (right panel) and passive walking (left panel).
(TIF)
