# Correction: Decoding Sensorimotor Rhythms during Robotic-Assisted Treadmill Walking for Brain Computer Interface (BCI) Applications

**DOI:** 10.1371/journal.pone.0157581

**Published:** 2016-06-09

**Authors:** The PLOS ONE Staff

The Data Availability statement for this paper is incorrect. The correct statement is: All relevant data are available via DANS (http://dx.doi.org/10.17026/dans-zcm-q8fq).
