# Supplementary material for: Decoding Sensorimotor Rhythms during Robotic-Assisted Treadmill Walking for Brain Computer Interface (BCI) Applications
Source: PLoS One. 2015 Dec 16;10(12):e0137910. doi: 10.1371/journal.pone.0137910 (PMC4686050; doi:10.1371/journal.pone.0137910)
Supplement: S1 File — (PDF) [file pone.0137910.s001.pdf]

Supporting Information

Accelerometer's data

Accelerometer data from the right leg was band-pass filter between 1 and 30Hz with a butterworth filter of second order. Gait cycles were determined according to the right heel strike, which in the accelerometer signal is seen as a vertical peak due to the deceleration. Peak searched was implemented in matlab according to the velocity of 1,5Km/h (for an example see S1 Fig.).

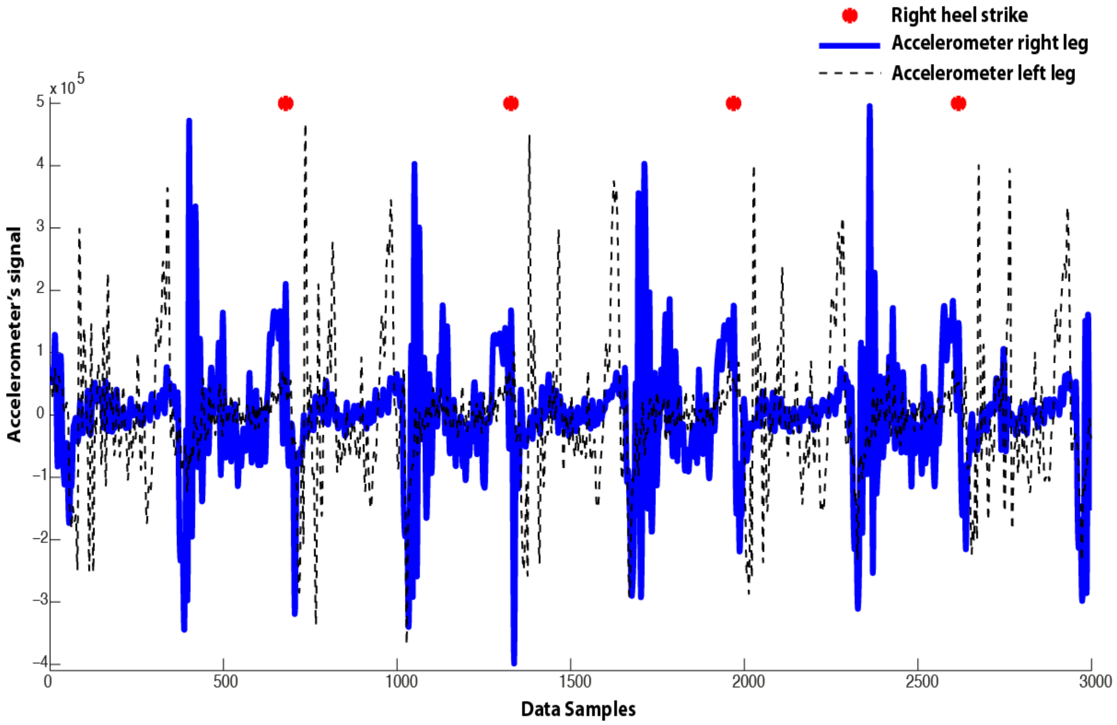

S1 Fig. Gait cycles were determined according to the right heel strike (red dots) using the accelerometer's data from the right (blue continue line) leg. The left leg's accelerometer data is as well illustrated (black dotted line) for comparisons.

EMG artifacts in EEG signals

By using canonical correlation analysis (CCA) the raw EEG signals were decomposed into EMG components and non-EMG components. For the purpose of extracting muscle artifacts from the EEG signals the EMG components were removed from the raw EEG data. However, in order to analyze the properties of the extracted EMG components, we removed the non-EMG components from the EEG raw data. The resulting signals were EEG signals highly contaminated by EMG.

A spectral analysis using Welch's method with a Hanning window of 250ms was performed in all the EEG channels for all four conditions (baseline before active walking, baseline before passive walking, active walking and passive walking). S2 Fig. illustrates the spectral analysis in all EEG electrodes for one of the participants. Clear muscular artifacts are shown in occipital, temporal and fronto-temporal electrodes, with a spectral strength stronger in higher frequencies (above 10Hz) than in the lower frequencies, which is a typical signature of EMG signals. Furthermore, the

ERD/S, calculated using equation (1), showed exactly the same pattern distributed in occipital, temporal and fronto-temporal electrodes, which differs from the ERD/S topographical pattern found from the non-EMG signals (i.e. strongest ERD over the Cz electrode in the foot motor area) (Fig. 2).

Furthermore, we performed the same classification analysis with a logistic-regression classifier on the EEG signals highly contaminated by EMG. We expected to find higher classification accuracies than when using the non-EMG components due the strong muscle artifacts present during walking.

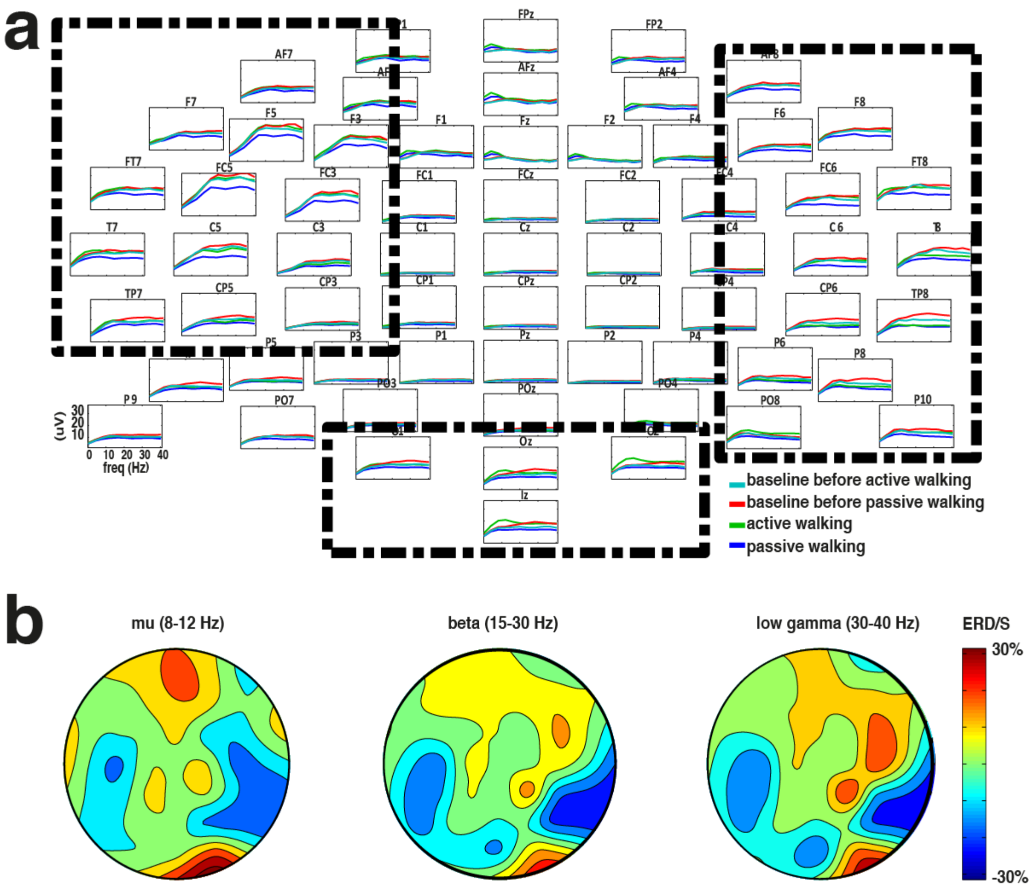

S2 Fig. Power density analysis of EMG artifacts in healthy volunteers. a. Spectral density analysis over all electrodes for active and passive walking and the baseline before passive and active walking conditions. b. Topographic distribution of event related desynchronization (ERD) and synchronization (ERS) in the mu (8-12 Hz), beta (15-30Hz) and low gamma (30-40Hz) bands.

The average classification accuracy was 93.6±8.0% (mean±std) when active walking was compared against baseline and 88.4±8.4% when passive walking was compared against baseline (S1 Table). A classification performance of 87.8±9.2% was achieved when active walking was compared against passive walking. Furthermore, when the baseline before active walking and the baseline before passive walking were compared the classification performance was 61.1±9.2% (close to chance level). In general, the classifier based on the EMG artifacts showed good performance, which indicates the need of using CCA to reject such components from the raw EEG signals.

S1 Table. Classification performance for different walking conditions (active and passive) and baseline.

participants	active walking vs baseline	passive walking vs baseline	active walking vs passive walking	baseline before active walking vs baseline before passive walking
**1**	98.7	89.3	95.2	58.3
**2**	97.4	94.9	90.6	73.2
**3**	100.0	100.0	87.0	68.8
**4**	94.8	92.0	85.1	56.0
**5**	100.0	82.9	92.7	78.1
**6**	95.1	94.2	72.2	56.3
**7**	98.6	85.9	94.7	60.0
**8**	88.6	85.8	92.8	54.6
**9**	88.3	89.9	96.6	48.8
**10**	74.1	69.3	71.0	57.4
**mean**	93.6	88.4	87.8	61.1
**std**	8.0	8.4	9.2	9.2

EEG temporal structure during the gait cycle

The EEG temporal structure in the centro-medial electrode Cz is shown in S3 Fig. for the raw data, EEG data filtered in the beta band (18 to 24 Hz), EEG data filter in the low gamma band (25 to 35 Hz) and event-related perturbations (ERSP) from 0 to 40Hz for one of the participants during active and passive walking.

In the raw EEG data a clear movement artifact is seen which is modulated according to the gait cycle for both passive and active walking in the Cz electrode.

For the beta and low gamma band a subtle modulation of the EEG amplitude is seen matching the modulations observed in the ERSP i.e. higher or lower absolute amplitude resulting in higher or lower amplitude in the spectrum, respectively.

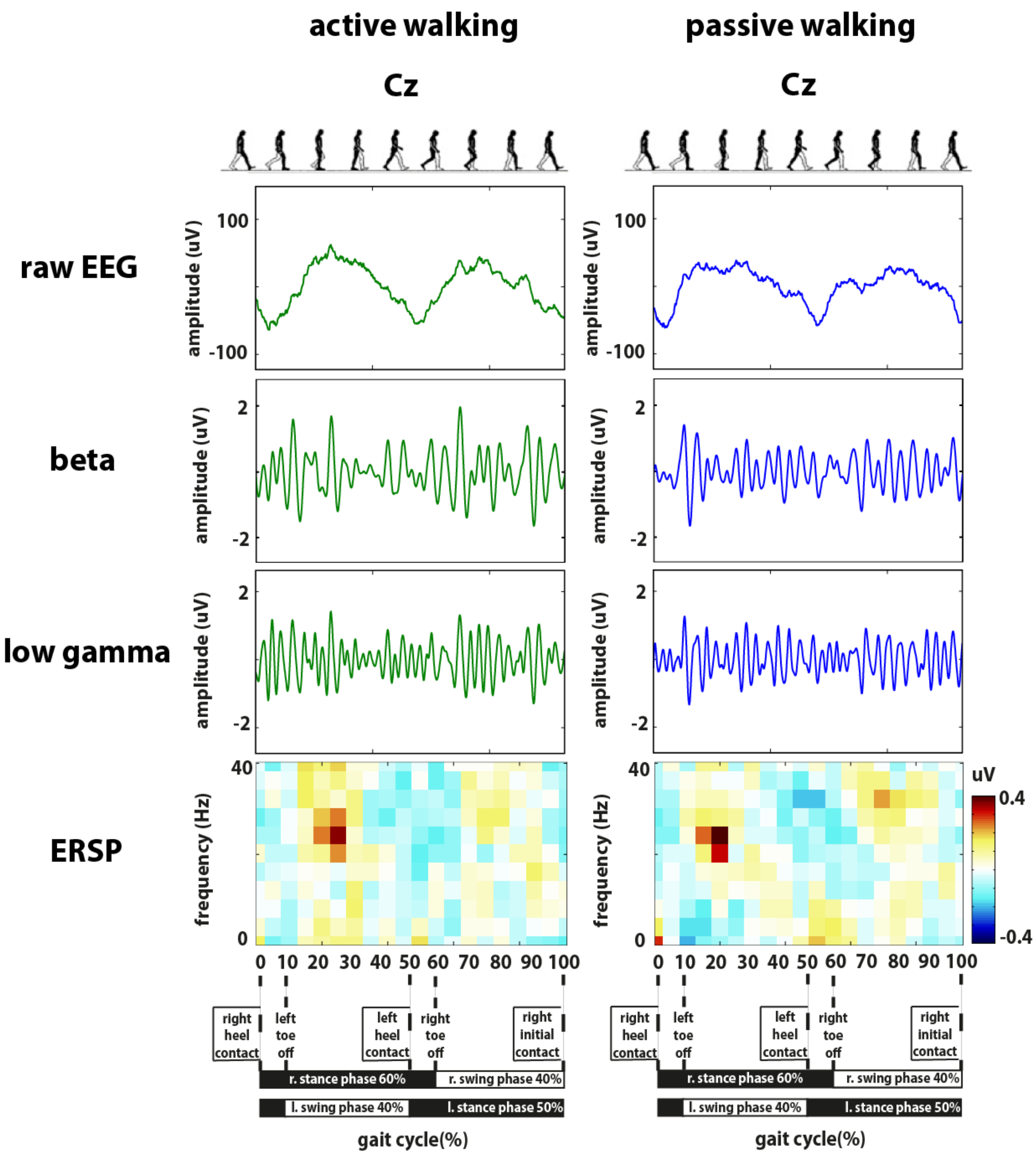

S3 Fig. EEG temporal structure (in Cz) during the gait cycle for one of the participants. From top to bottom: average across trials of raw data, EEG data filtered in the beta band (15-30 Hz), EEG data filter in the low gamma band (30-40 Hz) and event-related perturbations (ERSP) from 0 to 40Hz for active (right panel) and passive walking (left panel).
