# Supplementary figures and images for: Decoding Sensorimotor Rhythms during Robotic-Assisted Treadmill Walking for Brain Computer Interface (BCI) Applications
Source: PLoS One. 2015 Dec 16;10(12):e0137910. doi: 10.1371/journal.pone.0137910 (PMC4686050; doi:10.1371/journal.pone.0137910)

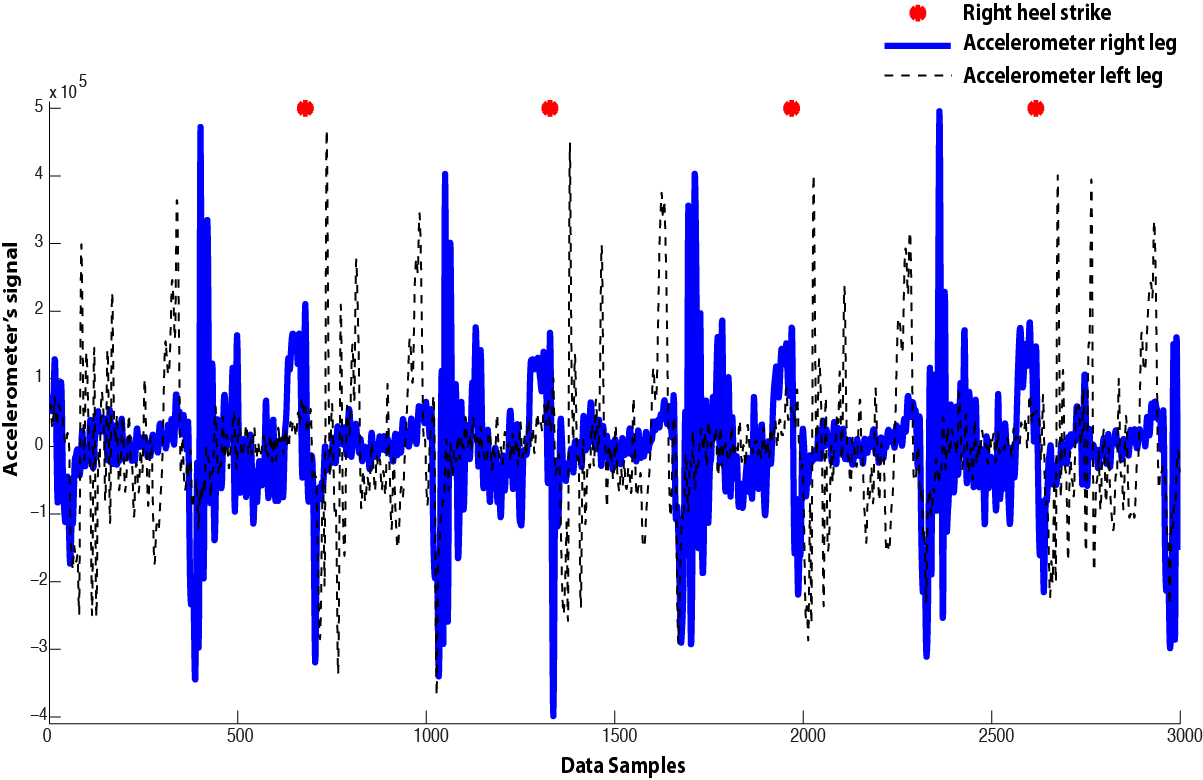

Supplement: S1 Fig — The left leg’s accelerometer data is as well illustrated (black dotted line) for comparisons. (TIF) [file pone.0137910.s002.tif]

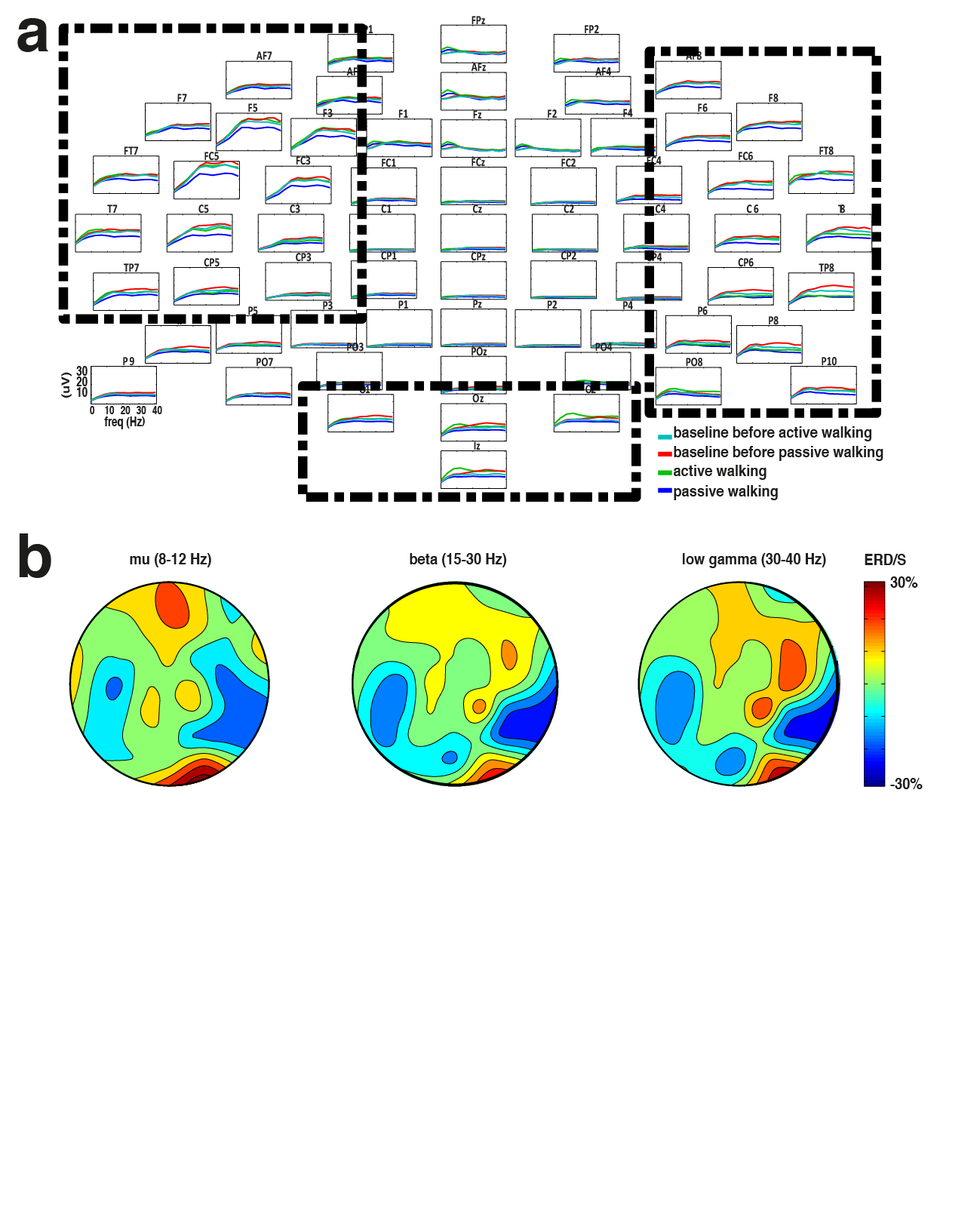

Supplement: S2 Fig — a. Spectral density analysis over all electrodes for active and passive walking and the baseline before passive and active walking conditions. b. Topographic distribution of event related desynchronization (ERD) and synchronization (ERS) in the mu (8–12 Hz), beta (15-30Hz) and low gamma (30-40Hz) bands. (TIF) [file pone.0137910.s003.tif]
